# Array tomography: trails to discovery

**DOI:** 10.1515/mim-2024-0001

**Published:** 2024-07-17

**Authors:** Kristina D. Micheva, Jemima J. Burden, Martina Schifferer

**Affiliations:** Department of Neurosurgery, Stanford University, Stanford, CA, 94305, USA; LMCB, University College London, London, UK; Center for Neurodegenerative Diseases (DZNE), Munich, Germany; Munich Cluster of Systems Neurology (SyNergy), Munich, Germany

**Keywords:** volume electron microscopy, array tomography, ATUM, light microscopy, ultramicrotomy, serial sectioning

## Abstract

Tissue slicing is at the core of many approaches to studying biological structures. Among the modern volume electron microscopy (vEM) methods, array tomography (AT) is based on serial ultramicrotomy, section collection onto solid support, imaging via light and/or scanning electron microscopy, and re-assembly of the serial images into a volume for analysis. While AT largely uses standard EM equipment, it provides several advantages, including long-term preservation of the sample and compatibility with multi-scale and multi-modal imaging. Furthermore, the collection of serial ultrathin sections improves axial resolution and provides access for molecular labeling, which is beneficial for light microscopy and immunolabeling, and facilitates correlation with EM. Despite these benefits, AT techniques are underrepresented in imaging facilities and labs, due to their perceived difficulty and lack of training opportunities. Here we point towards novel developments in serial sectioning and image analysis that facilitate the AT pipeline, and solutions to overcome constraints. Because no single vEM technique can serve all needs regarding field of view and resolution, we sketch a decision tree to aid researchers in navigating the plethora of options available. Lastly, we elaborate on the unexplored potential of AT approaches to add valuable insight in diverse biological fields.

## 1 Introduction

**Figure 1:**
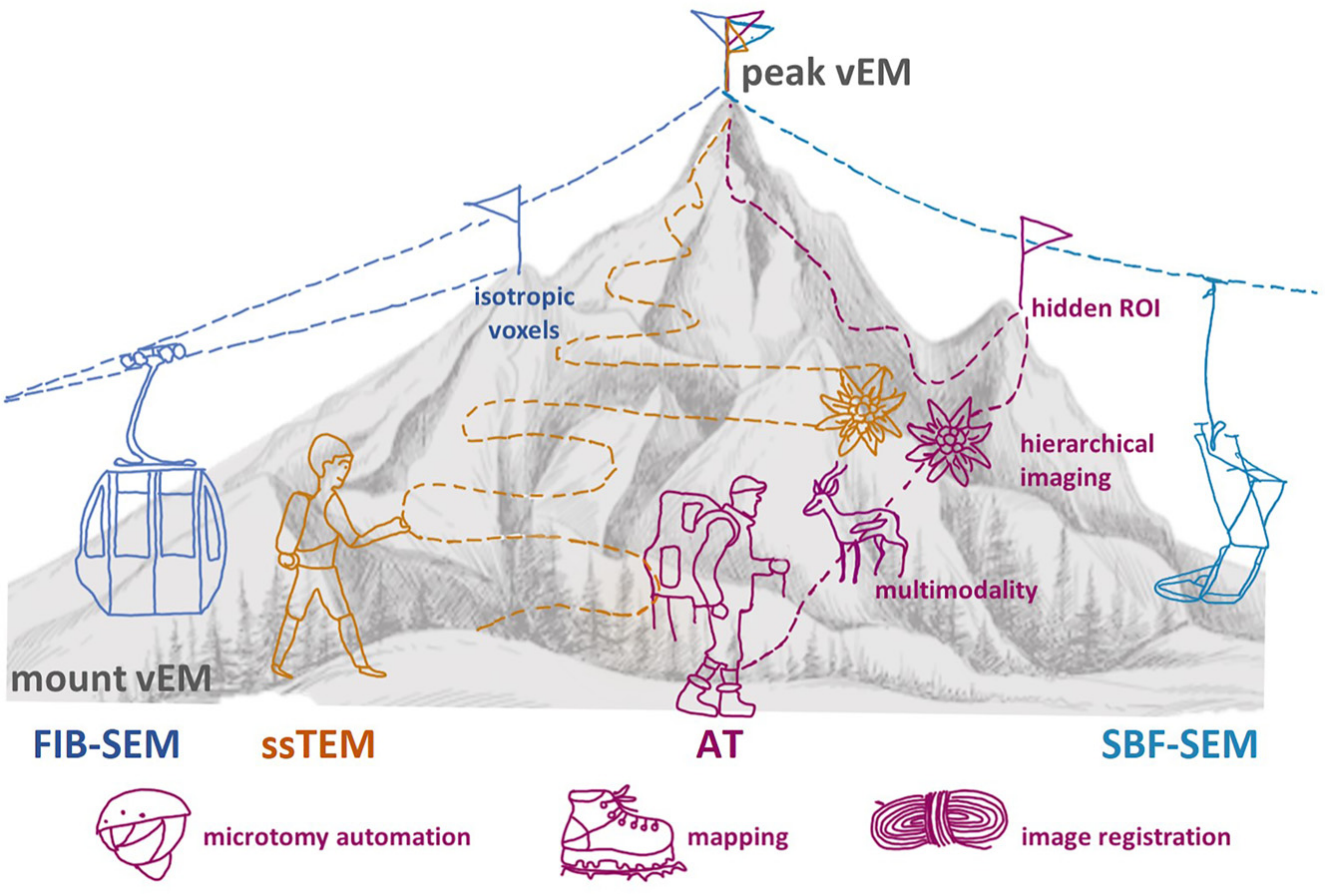
Illustration of different volume EM options. The colors indicate the different approaches: block-face (FIB-SEM, blue; SBF-SEM, cyan), serial section TEM (ssTEM, orange) and AT (magenta), including transport modes and trail trajectories. Improvements of the AT pipeline are shown as hiking equipment, symbolizing automation options as well as optimization of mapping and image registration. Hidden regions of interest (ROI) and completed volume ultrastructural data sets are peaks to be reached (flag) dependent on the approach. All in all, block-face methods are the fastest way to reach the top, albeit along a fixed route. Traditional ssTEM provides the highest level of detail, but along a challenging trail that is not for the faint of heart. The AT hiker is well equipped to climb any trail (shorter or longer) to reach the peak and explore treasures along the way, e.g. hierarchical imaging options (rare flowers) or multimodality (native animal species).

In the last few years, volume electron microscopy (vEM) has become part of the portfolio of a standard cell biological EM laboratory [1]. Among the most commonly used approaches are the block-face methods, that build on alternating sectioning and imaging of the remaining block-face within the microscope chamber, such as serial block-face SEM (SBF-SEM) [2] and focused ion beam scanning EM (FIB-SEM) [3]. Alternatively, for array tomography (AT) approaches serial sections are collected on a substrate, either on rigid support (rsAT) [4], or on tape using automated tape collecting ultramicrotomy (ATUM) [5], [6]. With either approach, the resulting images are aligned and reconstructed into a volume for further analysis and exploration. When designing new projects that require high resolution imaging, researchers have to choose the appropriate two- or three-dimensional EM technique among the available technical repertoire. This is often a complicated decision that has to take into account feasibility, scientific goals and economic aspects. Much like summiting a mountain, there are different ways to reach the destination, each with its advantages and drawbacks (Figure 1). Block-face techniques benefit from new commercial microscopy hardware solutions that are actively promoted, rather like a cableway, while AT builds on modular but basic workflows that can be accomplished using existing equipment, akin to a hiking adventure. Even though AT requires little investment, these approaches are still an underrepresented group of vEM solutions [7]. Experimentally, there is one major argument against the application of AT: because axial resolution is determined by section thickness, AT approaches usually result in anisotropic volumes. Yet, a vital advantage of AT is that section collection and preservation provide unique possibilities for repetitive, targeted and large area imaging. Samples can be inspected at different resolution regimes, a strategy known as hierarchical imaging, and with different modalities for a more comprehensive analysis. Barriers in favoring AT over other vEM techniques are the lack of know-how and a perception of AT as a laborious technique. For a novice in the field, usually this results in questions like “are the potential benefits worth the effort?”. Here, we point at possibilities to overcome these limitations and highlight automation options in order to professionally equip any aspiring “AT hiker”. We present a decision tree to aid the choice of the imaging approach for specific research projects. Lastly, we stress AT’s unique benefits and potential for further development.

## 2 Array tomography challenges and solutions

Originally, serial sectioning on grids for transmission EM (TEM) was mastered by a few gifted and patient imaging scientists [8], [9], [10], [11], [12]. The initial idea to automate serial section imaging by improving the laborious manual section collection onto grids was fueled by the “connectomics movement” which required the collection of thousands of serial sections [12]. With AT, section ribbons are picked onto a much larger and more stable surface, such as coverslip, wafer or tape, making it easier to collect long series of sections compared to picking up sections on tiny and fragile EM grids. Nevertheless, serial sectioning and collection remains a tedious step, and for many, a major obstacle in the pipeline. Sectioning and collecting thousands of 30–40 nm thick ultrathin sections is at the moment only possible in a few labs [13], [14], [15], however, collection of hundreds of 45–100 nm thick serial sections onto tape [16], [17], [18], or solid substrate [19], [20], [21], [22] is done routinely. There are various approaches to facilitate the collection of sections, with different laboratories having their own preferences. Serial section collection has spurred many low cost and unconventional solutions such as using hair spray to keep the sections together in a ribbon [23], deploying a fishing line to lift the substrate out of the knife boat [24], adapting a game controller to navigate the sectioned ribbon to the substrate [25], or using a paper clip to secure the substrate within the knife boat [22]. Commercial microtomy solutions include a manipulator handling the substrate [26], tape collectors [5], [27], big knife boats with water outlets [28] or modified ultramicrotomes (ARTOS 3D (Leica)). Even targeted trimming on a microtome, which is important for correlative work or for localizing a region of interest in a reference data set (usually generated by micro-computed tomography of a tissue block), has been automated [29] (UC Enuity, (Leica)). Further advancements like the usage of patterned silicon substrate with hydrophilic tracks for section ribbons [30] are expected to make ultramicrotomy and section collection for AT even more user friendly. A unique approach is the magnetic collection of sections (MagC) which uses magnetic resin and a diamond knife boat with a magnet to pack the sections onto the substrate [31]. Instead of trying to maintain section order during collection, MagC includes fluorescent beads in the resin and the gradual change of their pattern in the section series is used to determine section order. Recently, this magnet-driven approach has been further automated with the GAUSS-EM method [32]. The beauty and trade-off with magnetic approaches lies in the unordered collection which is easier to perform and results in a high tissue density per substrate area but comes with the cost of an additional ordering step in mapping and image alignment.

Another challenge in AT workflows is the mapping of the image series, i.e. identifying regions of interest on individual sections in order for serial acquisition, and image analysis. In comparison to block-face vEM methods which benefit from imaging the static, remaining block-face, AT generates sections with higher variability. Serial AT sections are often rotated relative to each other, especially when single, non-ribbon sections are collected on a tape, and they may have folds, dirt deposits or exhibit distortion due to compression artifacts [33], which complicates mapping and defining regions of interest for acquisition, as well as subsequent image alignment. AT is also a valuable method for imaging semithin sections, however in this case the higher difference in tissue features additionally affects image alignment. There are commercial mapping software packages for the respective SEM instruments (Maps (Thermo Fisher Scientific), Atlas (Zeiss), SEM Supporter (Jeol), ACAT (Hitachi)) and some pioneering labs use self-written scripts for SEM (WaferMapper: [34], [35], [36]), fluorescence (MosaicPlanner: [37], [38]) or combination of light and electron microscopy [39]. Some of these mapping solutions benefit from automated section detection that potentially fail for “difficult” samples (e.g. semithin sections), and others are designed for tedious but more flexible manual adaption and lack automation options (discussed in [33]). Similar to the mentioned mapping challenges, typical vEM image alignment pipelines are not directly applicable to AT data sets, as complex nonlinear deformations originating from folds, chatter, stretching or dirt deposition during sectioning have to be taken into account for reconstructing the correct biological structure [40]. However, conventional elastic alignment methods not only eliminate nonlinear deformations also but also may distort the natural biological morphology. This has been addressed by elastic registration [41], taking local continuity of neighboring sections into account [40], [42], [43]. A recently proposed solution builds on Siamese Encoding and Alignment by Multiscale Learning with Self Supervision (SEAMLeSS) [44]. An optimized SEAMLeSS alignment hierarchically (coarse to fine) applies self-supervised convolutional nets to compute dense correspondences between nearby images in combination with iterative fine-tuning [45]. Once more, the driving force for this improved alignment accuracy originates from the connectomics community.

## 3 Choice of imaging approach

Improvements and automation of serial sectioning and mapping, as well as image analysis, will decrease the entry barrier for the establishment of AT workflows. Unlike the block-face imaging techniques, where a more costly and specialised SEM is required (with either an in-vacuo microtome or a focused ion beam), the minimal instrumentation needed to process sample blocks by AT is already commonly available in many EM laboratories. Requiring only an ultramicrotome and a diamond knife, as well as a fluorescence microscope and/or a SEM, AT workflows are cost effective approaches accessible to many. This is preferentially complemented by mapping software, a collecting device or facilitator and a sputter coater. AT-specific consumables mainly comprise the collecting support material and depend on the choice of AT approach, rsAT (solid, e.g. silicon or glass) versus ATUM (tape). Given proper training and availability of AT equipment, a scientist or EM facility manager will have to decide on the appropriate AT or other vEM technique to apply to each project [46]. Some projects even require taking one step back to decide if an EM approach is required or the research question can be better answered using light microscopy. There are currently a variety of choices to interrogate intact samples with high- or super-resolution light microscopy, such as confocal, single-molecule localization, expansion microscopy, for which we refer the reader to several excellent recent reviews [47], [48], [49]. Among all options that AT offers, one can consider immunofluorescence AT with the benefit that it can be combined with conjugate EM on the exact same physical sections, thus allowing ultrastructural confirmation of the light microscopy analysis.

**Figure 2:**
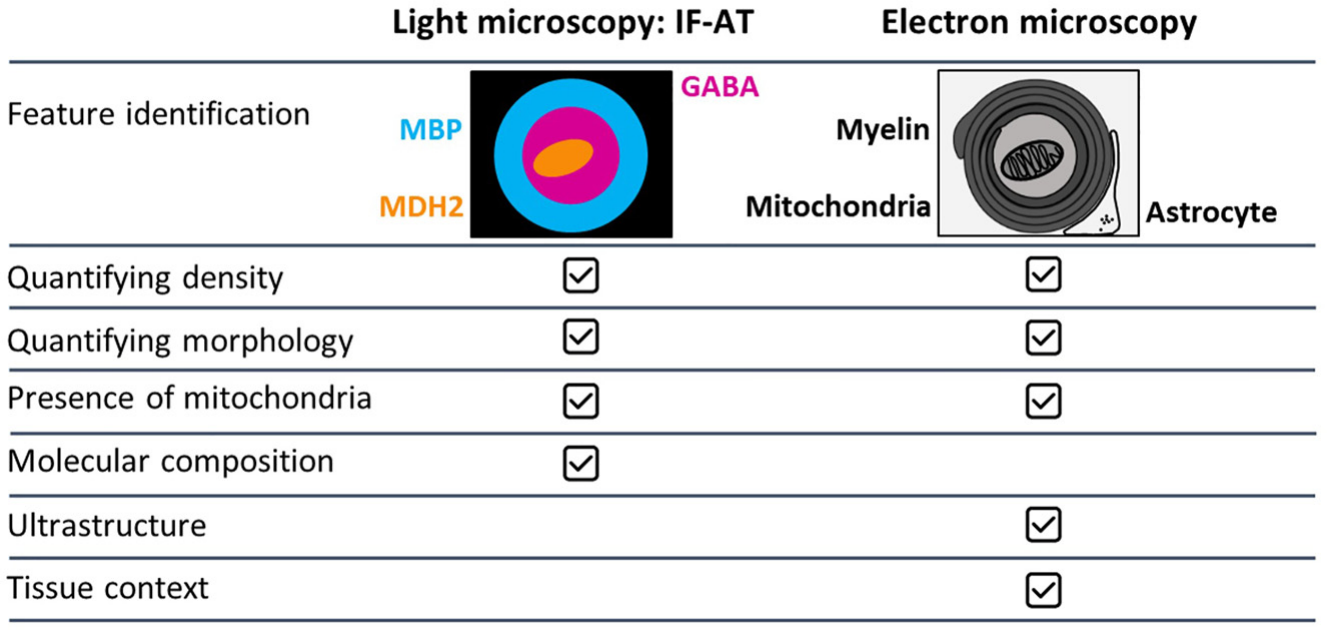
Immunofluorescence AT (IF-AT) versus EM. Both IF-AT and EM can be used to study myelinated axons in the mammalian brain [55]. With IF-AT, myelin is readily identified using myelin basic protein (MBP) immunolabeling, and mitochondria using MDH2 immunolabeling. With EM, these features are defined by their distinct ultrastructural appearance. Both approaches can quantify the prevalence and distribution of myelin within the tissue, as well as basic morphology. IF-AT can further analyze the molecular composition of these axons (e.g. neurotransmitter content, GABA) and their myelin, while EM provides the ultrastructural information to assess myelin or axonal integrity, as well as the tissue context to examine features such as the relationship between myelin and astrocytic processes. IF-AT and EM can be applied together as conjugate AT when both molecular and ultrastructural information is desired [37]. The method of choice varies depending on the scientific question.

Immunofluorescence AT [4], [25] represents a volume light microscopy method which falls in a somewhat unique space between conventional immunohistochemistry and EM, and this means that it is often overlooked by practitioners of either method. Immunofluorescence AT samples are prepared in a similar way as samples for EM, by embedding in resin and ultrathin serial sectioning, and require tissue processing expertise and ultramicrotomes typical of EM labs. Once sectioned, however, the tissue is labeled with immunofluorescence and imaged on a fluorescence microscope. Because of the thinness of sections (45–100 nm), immunofluorescence AT has much higher lateral and axial resolution compared to conventional immunofluorescence on thicker (tens of µm) sections. There is no out-of-focus fluorescence on immunolabeled ultrathin sections, and the sections are imaged at ideal conditions as they are mounted directly on the coverslip, which brings the lateral resolution to the theoretical maximum, around 200 nm. The z-resolution is higher and is determined by the thickness of the section, which in practice means that an AT section imaged by a widefield fluorescence microscope will provide an order of magnitude higher *z*-resolution compared to a much more expensive confocal microscope imaging of a conventional immunofluorescence sample. The x-y resolution can be further improved by applying deconvolution [50], or by imaging the sections with super-resolution methods like structured illumination, STED or STORM [51], [52], [53]. Furthermore, immunofluorescence AT enables the use of multiple antibodies to characterize dozens of antigens on the same or adjacent sections. Depending on the research question and especially when the studied feature is large enough to be definitively resolved (e.g. larger than 200 nm in diameter), immunofluorescence AT can be a viable and more efficient alternative compared to EM. The crucial element for its success is the availability of suitable antibodies to reliably label the features of interest in the resin-embedded sections [52], [54], as well as antibodies against other reference tissue components to provide tissue context (Figure 2). For example, the smallest myelinated axons in the central nervous system are around 200 nm in diameter and there are excellent antibodies that label myelin for AT, allowing a detailed investigation of the abundance and trajectories of these axons [55]. Most, but not all mammalian synapses are also larger than 200 nm, and immunofluorescence AT has been used for synapse quantification, including analysis of changes associated with disease of the human brain [56], [57].

**Figure 3:**
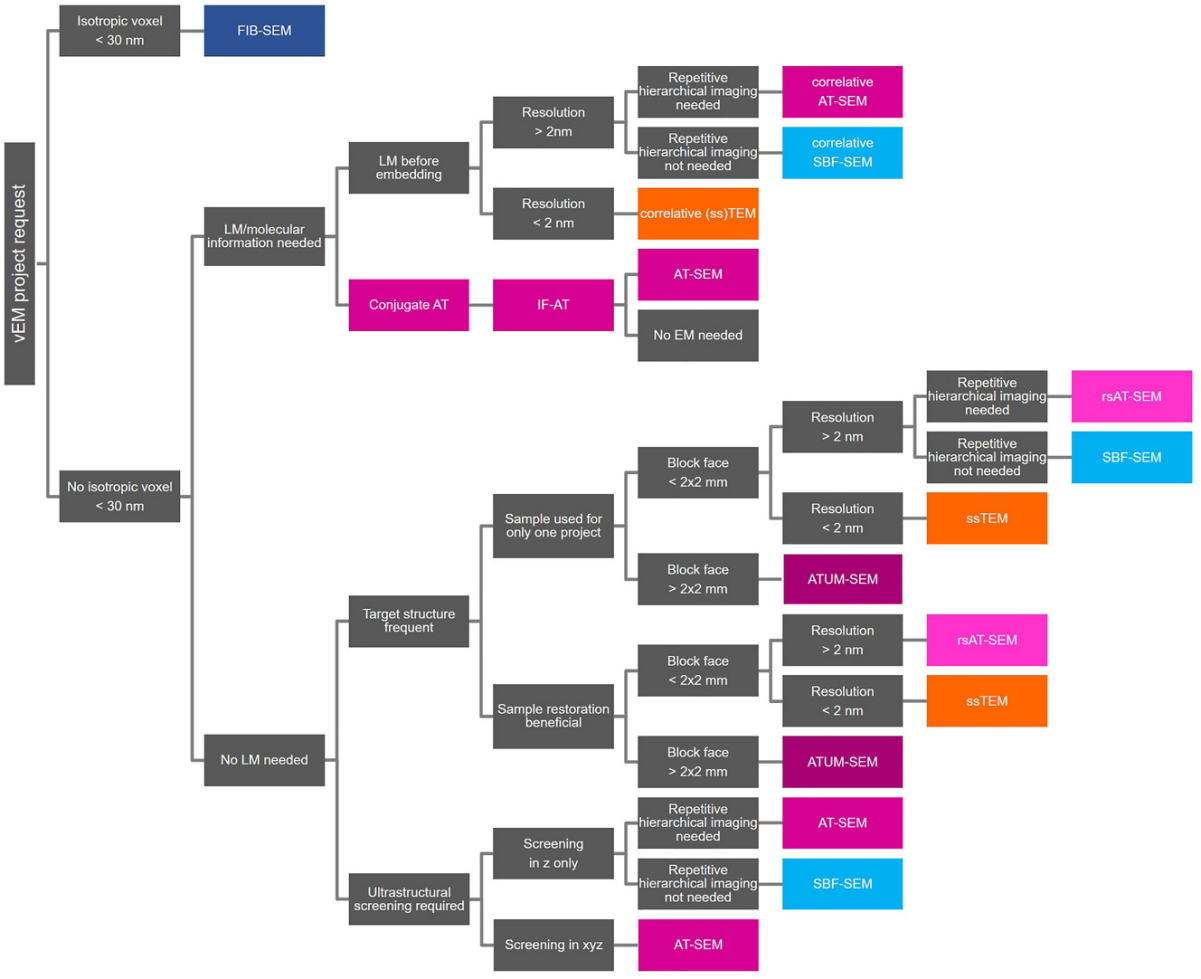
Proposed flow chart to support decision making between different vEM approaches for life science EM facilities with standard equipment. While there are physical constraints that make some techniques more suitable for specific research projects, the boundaries are quite flexible. Decision making is further impacted by technology accessibility and capacity, available expertise, and costs. In many cases, different approaches are equally well-suited and we only give suggestions for decision making. As an example, the criterium “sample restoration beneficial” refers to the advantage of methods that preserve the imaged sections, like AT or ssTEM, in case of precious samples (e.g. human biopsy or a tissue from a complex mouse model) that will potentially be used to answer several present and future scientific questions. Samples can be reimaged at different resolution regimes at a later time point, and shared around the world for alternative investigations. In case this is not needed, it might be more straightforward to apply block-face techniques. SBF-SEM provides restricted but not a “repetitive hierarchical imaging” option through the option to image at different resolution regimes during a run. In this chart we distinguish AT techniques according to their support material for section collection: rigid support AT (rsAT) and tape (ATUM) as this influences the block-face size and shape; when not specified, e.g. AT-SEM, either support material can be used. Scanning electron microscopy (SEM), transmission electron microscopy (TEM), serial section TEM (ssTEM), focused ion beam (FIB), serial block-face (SBF), rigid support AT (rsAT), automated tape collecting ultramicrotomy (ATUM), correlated light and electron microscopy (CLEM), region of interest (ROI).

Immunofluorescence AT, however, cannot provide the rich ultrastructural detail and subnanometer resolution provided by EM. For such projects, EM is the obvious approach. With EM, there are many different routes to take and more decisions to make to choose the appropriate technique. When two-dimensional EM is sufficient, the choice is between AT and TEM, and if vEM is required, then block-face techniques become a major contender. We developed a simplified decision tree to help guide the choice of EM imaging approach depending on the requirements of different research projects (Figure 3).

Until recently, two-dimensional EM has informed much of our understanding of tissue ultrastructure or subcellular localization of proteins. When volume information is not required, AT can be considered versus two-dimensional TEM in cases where lateral pixel size of approximately 2–3 nm is sufficient. AT would be preferred if large areas have to be screened for target identification or if quantification of multiple regions or different samples is needed. Sections from many samples can be mounted on the same substrate, and AT mapping software not only covers serial section acquisition but can be used to set up an imaging run for multiple ROIs very easily, similar to SerialEM for TEM [58]. While there is no limitation regarding tissue area size from an imaging perspective, a bottleneck from an ultramicrotomy point of view remains. Currently, consistent sectioning with ATUM of block-face sizes as large as 5.16 by 3.67 mm has been reported [59], and there is potential to obtain even larger areas using long knife edges (8 mm, diatome) (Figure 3). The imaging of such large areas is facilitated by fast large area scanning (MultiSEM (Zeiss), Fast EM (Delmic)).

Similarly to two-dimensional cases, the resolution is a major factor for deciding on a vEM approach. TEM, the slowest of all “trails” to the vEM summit (Figure 1), is generally preferred when subnanometer resolution is required, for example to identify the electrical connections between neurons – the gap junctions [60]. For biological samples, SEM resolution is limited by the probe size which determines the scanned pixel size. A field emission source can generate a much smaller probe size compared to a standard tungsten filament, and thus a field-emission SEM is required for most AT applications. TEM provides higher resolution options with standard detectors, mostly limited by staining intensity of the sample. The benefits of TEM, however, come at the cost of much more tedious serial section collection and handling, and a heightened risk of losing individual sections due to small and fragile support substrates. For larger section sizes and volumes, customized TEMs and software are used [14], [61], [62]. Compared to TEM, block-face SEM techniques are highly automated, much more user friendly, and faster [63]. SBF-SEM is appropriate for less resolution-demanding projects, especially in cases where multiple volumes have to be imaged and analyzed. FIB-SEM enables imaging at isotropic voxels, but with higher time cost and much more limited block-face size (0.1 × 0.1 mm) [64], compared to SBF-SEM imaging (2 × 2 mm). A novel variant, plasma FIB-SEM (pFIB-SEM) provides alternative milling gases, thereby enabling faster runs and larger block faces [65]. Block-face methods require that all tissue labeling be done before sectioning and new approaches are facilitating consistent labeling and reagent penetration for larger samples [66], [67]. However, block-face approaches do not preserve the sections after they are imaged and therefore do not allow repeated imaging at a different resolution or with a different modality, which both TEM and AT offer.

## 4 Array tomography’s unique features and potential

A major advantage of AT compared to block-face techniques is its restorative character, because the arrays of sections are preserved after imaging. Assembled AT samples can be stored for long periods of time (years) and can be reimaged at different resolutions and on different instruments. This feature, combined with the large field of view, is especially helpful for hierarchical imaging, when searching for rare events because the sections can be imaged at low resolution first to locate the feature of interest, which is then imaged at high resolution [28], [68]. Conversely, having imaged the region of interest at high magnification, any particularly interesting structures can be reimaged with lower resolution or different fields of view, thus capturing wider environmental contexts that are informative to the research. Additionally, the samples become a useful resource for the future and can be interrogated again by the same or different researchers to look at different regions or ask different questions. Moreover, different modalities can be applied on the same sections. For example, immunofluorescence AT can be followed by SEM imaging of the ultrastructure in conjugate AT [37]. Both imaging modalities in this case are performed on the exact same physical sections which removes axial ambiguities during image registration, in contrast to most CLEM methods where light microscopy is performed on the intact sample before sectioning for EM imaging. There are, however, several CLEM approaches that enable the light microscopic interrogation of resin-embedded samples, either before sectioning [69], or after sectioning, with alternating sections used for light and TEM [70], including ssTEM [71].

AT allows a unique flexibility of scales in the axial dimension, by variation of section thickness and the imaging of every other section. SEM imaging can be performed on ultrathin sections (down to 30 nm) to obtain ultrastructural details [15], as well as on semithick sections (up to 1 μm and above) for fast imaging of large volumes with cellular detail [72]. When choosing the specific 3D approach for a given project, an important consideration is the size of the sample. ATUM is much more convenient for large samples, because the tape can accommodate an unrestricted number of even large sections, while solid substrates have a much more limited area to work with. However, if fluorescence imaging of the samples is also desired, then transparent solid substrates such as glass coverslips are preferred.

Besides the many advantages of the AT approach, there is still dormant potential including the modular character which has not been fully exploited. AT consists of separate distinct steps or “modules” (sample preparation, collection, labeling, mounting, imaging, image analysis), that can be combined in different ways. The different steps of the AT pipeline can be performed at different time points, shared among staff or between geographically distinct facilities with complementing expertise or equipment. The sample block, the section series on support or the image series can be shipped or transferred globally. This will favor the strengthening of vEM imaging in locations without access to all the necessary equipment and will enable scientists around the world to address their unique imaging needs in diverse biological fields such as pathobiology, microbiology or botany [73]. AT’s “tissue library” character enables practically indefinite storage of precious samples like human biopsies or autopsies and their reimaging at different resolutions and on different instruments [15], [74]. Besides reimaging with electron or light microscopy and in order to further extract molecular readouts, promising first attempts of correlating spatial transcriptomics with ultrastructural data obtained by AT are emerging [75]. Moreover, semithin and semithick sectioning on solid support material enable the combination of AT and block-face techniques [76], [77], [78], benefiting from the advantages of both approaches. While AT provides fast screening for a biological target, it can subsequently be imaged using FIB-SEM or gas cluster ion beam (GCIB) [79] at isotropic voxels. Consequently, AT type of methods can be combined to tap into different resolution regimes. Recently, the AT principle was even applied to develop a serial cryo-EM approach called Serial Lift-Out [80] that similarly provides access to high-resolution ultrastructural details at different scales. Thus, AT’s modularity not only enables sharing of samples for multimodal, correlative and repeated microscopy but will allow the exploitation of integrated imaging data by different scientific groups around the globe. This development will be increasingly relevant with the progress of deep learning tools for microscopy. In order to make AT accessible to many researchers, we are assembling relevant information, protocols, resources and publications in an AT dedicated webpage (www.arraytomography.org), preparing a hands-on annual workshop for AT, and bringing AT-interested scientists together online in a focused interest group within the volume EM community (https://www.volumeem.org/at-fig.html).

## 5 Conclusions

AT techniques continue to evolve, including section collection automation, combination with light microscopy super resolution techniques [53] or other EM techniques [77], [78], as well as image analysis. For a novice in the field, both the decision of the best technical approach and its execution can present significant barriers to reach the summit, but the many anticipated rewards and surprise discoveries along the trail make the AT approach worth investing the effort. While personal training of disseminators is crucial [73], [81], the creation of an international network of AT imaging scientists will facilitate optimization and further advances of AT workflows. The imaging community will highly benefit from cross-modal and interdisciplinary collaborations as well as global integration of AT modules and thereby help to advance diverse biological fields.
